# Lipoblastoma Mimicking Inguinal Hernia

**Published:** 2015-01-01

**Authors:** Sevgi Buyukbese Sarsu, Suleyman Cuneyt Karakus, Burcu Belen

**Affiliations:** 1 Department of Pediatric Surgery, Gaziantep Children’s Hospital, Turkey.; 2 Department of Pediatric Hematology and Oncology, Gaziantep Children’s Hospital, Turkey.

**Dear Sir,**

Lipoblastoma is a rare benign soft tissue tumor occurring mostly in infancy and early childhood. It arises from white embryonic fat. Adipose tissue tumors constitute about 6% of all soft tissue neoplasms in children and lipoblastoma accounts for only 5 to 30% of adipose tissue tumors.[1-3] Many patients present with a painless mass, which lead to misdiagnosis. A 2-year old girl was admitted with an asymptomatic progressively enlarging swelling at the right inguinal region for 4 months. There were no obstructive bowel symptoms. She had a history of right inguinal hernia repair 8 months ago (operative notes were not available). On physical examination, a non-tender, mobile and irreducible (2x3 cm) swelling was noted. Inguinal ultrasonography was normal. Patient underwent surgical exploration of the swelling through the old scar with the initial diagnosis of recurrent sliding inguinal hernia of ovary. At operation, there was no recurrence of inguinal hernia and the inguinal swelling was more like a mass. The tumor was completely excised, with the resulting specimen of 4 x 2.5 x 2cm, with no invasion into the surrounding tissues. The histopathological examination revealed lobulated tissue composed of immature fat cells separated by fibro-vascular septa and areas with a myxoid matrix. The lobules contained lipoblasts in different stages of differentiation, ranging from primitive, spindle-shaped cells to lipoblasts simulating mature fat cells (Fig.1). She has been followed up every 3 months for 2 years and no recurrence has occurred.

**Figure:**
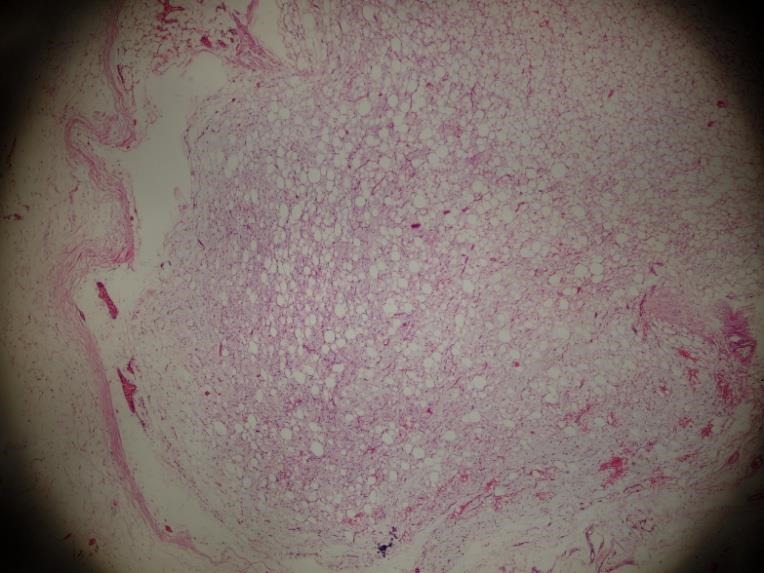
Figure 1:Histopathologically predominantly vacuolated lipoblasts mixed with spindled cells, with a fibrous capsule noted.

Lipoblastomatous tumors are rare, benign adipose tissue tumors of infancy and childhood. Ninety percent of reported cases are under the age of three years and 55% less than 1 year of age.[3] Histopathologically, these tumors are classified into two forms as lipoblastoma (circumscribed type) and lipoblastomatosis (diffuse, multicentric and infiltrative type).[4] The inguinal region is an uncommon location for this tumor.[2] Because of the nonspecific presentation of lipoblastomas, they are often mistaken for other conditions.[5] The previous operation on the same side may point to a possible recurrence, however, the history and physical examination did not correlate with recurrence as it was a progressive swelling and was of significant size. Ultrasound which was reported as normal could have differentiated between a fatty mass and a firm structure like ovary, however, this modality could not help us. Patients with lipoblastoma usually have a good prognosis despite the tumor's potential to invade locally and grow rapidly.[2] Post-operative follow-up is important to detect relapses but appropriate length of follow-up is controversial.

## Footnotes

**Source of Support:** Nil

**Conflict of Interest:** None declared

